# Supplementary material for: Mapping the global geography of cybercrime with the World Cybercrime Index
Source: PLoS One. 2024 Apr 10;19(4):e0297312. doi: 10.1371/journal.pone.0297312 (PMC11006133; doi:10.1371/journal.pone.0297312)
Supplement: S1 Indices — Full indices for the WCI Overall and each WCI Type. (PDF) [file pone.0297312.s001.pdf]

S1 Indices. WCI indices

Full indices for each type of cybercrime (WCI_{type}), as well as the full Overall index ($WCI_{overall}$), are shown below (Tables S1-S6). Each index includes the rank, number of nominations, scores for impact (I), professionalism (P) and technical skill (TS), overall score, and WCI Score for each country. Each index is ordered and ranked by WCI Score and includes all 197 UN-recognised nations. Countries that were not nominated are listed at equal null ranking, in alphabetical order. Indices below are shown in the order in which the cybercrime types appeared in the survey.

Legend: Noms = Nominations ; I = Impact ; P = Professionalism ; TS = Technical skill ; Overall = average of I, P, and TS. I, P, TS, and Overall are scored out of 10. WCI Score is scored out of 100.

Table S1. World Cybercrime Index - Overall

Rank	Noms	Country	I	P	TS	Overall	WCI Score
1	304	Russia	8.96	8.81	8.71	8.84	58.39
2	202	Ukraine	8.37	8.29	8.24	8.30	36.44
3	162	China	8.22	7.70	7.81	7.91	27.86
4	154	United States	7.99	7.21	7.21	7.47	25.01
5	143	Nigeria	8.25	6.49	5.80	6.85	21.28
6	96	Romania	7.12	7.04	7.15	7.10	14.83
7	65	North Korea	7.91	7.23	7.38	7.51	10.61
8	57	United Kingdom	7.86	7.21	6.75	7.27	9.01
9	63	Brazil	6.90	6.35	6.32	6.52	8.93
10	40	India	7.90	6.60	6.65	7.05	6.13
11	33	Iran	6.88	6.45	6.64	6.66	4.78
12	25	Belarus	6.84	7.20	7.32	7.12	3.87
13	23	Ghana	8.57	6.83	6.09	7.16	3.58
14	20	South Africa	6.95	5.35	5.50	5.93	2.58
15	16	Moldova	7.38	7.19	7.56	7.38	2.57
16	14	Israel	8.43	8.21	8.07	8.24	2.51
17	13	Poland	8.46	7.92	7.15	7.85	2.22
18	13	Germany	8.00	7.54	7.54	7.69	2.17
19	12	Netherlands	7.75	7.25	7.08	7.36	1.92
20	11	Latvia	6.73	7.36	7.00	7.03	1.68
21	11	Kazakhstan	7.09	6.91	7.00	7.00	1.67
22	12	Vietnam	7.08	5.92	5.33	6.11	1.59
23	10	Estonia	6.80	7.60	7.50	7.30	1.59
24	10	United Arab Emirates	7.90	6.70	6.80	7.13	1.55
25	8	Canada	8.00	7.38	7.75	7.71	1.34
26	12	Malaysia	6.33	4.58	4.33	5.08	1.33
27	11	Philippines	6.27	5.00	4.27	5.18	1.24
28	10	Turkey	5.50	5.70	5.60	5.60	1.22
29	8	France	6.50	6.62	7.00	6.71	1.17
29	8	Indonesia	6.88	6.88	6.38	6.71	1.17
31	8	Bulgaria	6.62	5.88	6.00	6.17	1.07
32	7	Thailand	7.43	6.57	5.71	6.57	1.00
33	7	Mexico	7.14	6.14	6.00	6.43	0.98
34	8	Australia	6.50	5.00	4.88	5.46	0.95
35	5	Italy	7.40	7.40	7.40	7.40	0.80
36	5	South Korea	7.40	7.40	7.00	7.27	0.79
37	5	Cameroon	8.20	5.80	5.40	6.47	0.70
37	5	Colombia	7.20	6.60	5.60	6.47	0.70
39	4	Gambia	9.50	6.00	6.00	7.17	0.62
40	4	Czech Republic	8.00	7.25	5.00	6.75	0.59
41	4	Switzerland	6.75	6.50	5.75	6.33	0.55
42	3	Cyprus	8.33	7.67	8.00	8.00	0.52
43	3	Myanmar	9.67	9.00	5.00	7.89	0.51
44	4	Pakistan	6.75	5.25	5.50	5.83	0.51
45	4	Sierra Leona	8.75	4.00	4.50	5.75	0.50
46	3	Laos	9.33	9.33	4.00	7.56	0.49
47	3	Haiti	8.67	6.67	6.67	7.33	0.48
48	4	Morocco	6.75	4.75	4.00	5.17	0.45
49	3	Panama	7.00	7.33	6.33	6.89	0.45

50	3	Belize	7.33	6.67	6.33	6.78	0.44
51	3	Cambodia	9.00	8.33	2.00	6.44	0.42
52	2	Armenia	9.50	9.50	9.50	9.50	0.41
53	2	Lithuania	9.00	8.50	9.00	8.83	0.38
54	3	Chile	6.67	5.67	5.00	5.78	0.38
55	3	Ecuador	6.33	5.67	4.67	5.56	0.36
56	4	Ivory Coast	5.25	4.00	2.75	4.00	0.35
56	2	Afghanistan	9.50	7.50	7.00	8.00	0.35
56	2	Angola	8.50	6.50	9.00	8.00	0.35
59	3	Peru	6.33	5.33	4.33	5.33	0.35
60	3	Algeria	6.67	4.67	4.33	5.22	0.34
61	2	Lebanon	8.00	8.00	7.00	7.67	0.33
62	2	Seychelles	8.50	7.00	7.00	7.50	0.33
63	2	Benin	8.00	6.00	7.50	7.17	0.31
63	2	Kenya	9.00	6.50	6.00	7.17	0.31
65	3	Venezuela	5.67	4.00	4.00	4.56	0.30
66	2	Georgia	6.00	6.50	6.00	6.17	0.27
66	2	Spain	6.50	6.00	6.00	6.17	0.27
68	2	Senegal	9.50	3.50	4.50	5.83	0.25
69	2	Tunisia	7.00	4.50	4.00	5.17	0.22
70	2	Slovenia	5.50	5.00	4.50	5.00	0.22
71	1	Sweden	9.00	10.00	10.00	9.67	0.21
72	1	Cuba	9.00	9.00	9.00	9.00	0.20
72	1	Mozambique	10.00	8.00	9.00	9.00	0.20
74	1	Greece	10.00	8.00	8.00	8.67	0.19
74	1	Uganda	10.00	7.00	9.00	8.67	0.19
76	1	Belgium	9.00	8.00	8.00	8.33	0.18
76	1	Togo	10.00	10.00	5.00	8.33	0.18
78	1	Malta	8.00	8.00	8.00	8.00	0.17
78	1	Mauritius	9.00	7.00	8.00	8.00	0.17
80	1	Equatorial Guinea	10.00	8.00	5.00	7.67	0.17
81	1	Hungary	7.00	7.00	7.00	7.00	0.15
81	1	Serbia	7.00	7.00	7.00	7.00	0.15
83	1	Argentina	6.00	7.00	7.00	6.67	0.14
83	1	Jamaica	9.00	7.00	4.00	6.67	0.14
85	1	Bosnia and Herzegovina	5.00	7.00	7.00	6.33	0.14
85	1	Central African Republic	7.00	7.00	5.00	6.33	0.14
87	2	Botswana	3.00	3.00	3.00	3.00	0.13
87	2	Ethiopia	3.00	3.00	3.00	3.00	0.13
87	2	Zambia	3.00	3.00	3.00	3.00	0.13
87	1	Azerbaijan	9.00	6.00	3.00	6.00	0.13
87	1	Dominican Republic	9.00	7.00	2.00	6.00	0.13
87	1	Luxembourg	6.00	7.00	5.00	6.00	0.13
93	1	Japan	5.00	4.00	6.00	5.00	0.11
94	1	Mali	10.00	1.00	2.00	4.33	0.09
94	1	Syria	3.00	5.00	5.00	4.33	0.09
96	1	Guinea-Bissau	10.00	1.00	1.00	4.00	0.09
97	1	Egypt	3.00	3.00	5.00	3.67	0.08
98	NA	Albania	NA	NA	NA	NA	NA
98	NA	Andorra	NA	NA	NA	NA	NA
98	NA	Antigua and Barbuda	NA	NA	NA	NA	NA
98	NA	Austria	NA	NA	NA	NA	NA

98	NA	The Bahamas	NA	NA	NA	NA	NA
98	NA	Bahrain	NA	NA	NA	NA	NA
98	NA	Bangladesh	NA	NA	NA	NA	NA
98	NA	Barbados	NA	NA	NA	NA	NA
98	NA	Bhutan	NA	NA	NA	NA	NA
98	NA	Bolivia	NA	NA	NA	NA	NA
98	NA	Brunei	NA	NA	NA	NA	NA
98	NA	Burkina Faso	NA	NA	NA	NA	NA
98	NA	Burundi	NA	NA	NA	NA	NA
98	NA	Cape Verde	NA	NA	NA	NA	NA
98	NA	Chad	NA	NA	NA	NA	NA
98	NA	Comoros	NA	NA	NA	NA	NA
98	NA	Congo, Dem. Republic	NA	NA	NA	NA	NA
98	NA	Congo, Republic	NA	NA	NA	NA	NA
98	NA	Cook Islands	NA	NA	NA	NA	NA
98	NA	Costa Rica	NA	NA	NA	NA	NA
98	NA	Croatia	NA	NA	NA	NA	NA
98	NA	Denmark	NA	NA	NA	NA	NA
98	NA	Djibouti	NA	NA	NA	NA	NA
98	NA	Dominica	NA	NA	NA	NA	NA
98	NA	East Timor	NA	NA	NA	NA	NA
98	NA	El Salvador	NA	NA	NA	NA	NA
98	NA	Eritrea	NA	NA	NA	NA	NA
98	NA	Eswatini	NA	NA	NA	NA	NA
98	NA	Fiji	NA	NA	NA	NA	NA
98	NA	Finland	NA	NA	NA	NA	NA
98	NA	Gabon	NA	NA	NA	NA	NA
98	NA	Grenada	NA	NA	NA	NA	NA
98	NA	Guatemala	NA	NA	NA	NA	NA
98	NA	Guinea	NA	NA	NA	NA	NA
98	NA	Guyana	NA	NA	NA	NA	NA
98	NA	Honduras	NA	NA	NA	NA	NA
98	NA	Iceland	NA	NA	NA	NA	NA
98	NA	Iraq	NA	NA	NA	NA	NA
98	NA	Ireland	NA	NA	NA	NA	NA
98	NA	Jordan	NA	NA	NA	NA	NA
98	NA	Kiribati	NA	NA	NA	NA	NA
98	NA	Kuwait	NA	NA	NA	NA	NA
98	NA	Kyrgyzstan	NA	NA	NA	NA	NA
98	NA	Lesotho	NA	NA	NA	NA	NA
98	NA	Liberia	NA	NA	NA	NA	NA
98	NA	Libya	NA	NA	NA	NA	NA
98	NA	Liechtenstein	NA	NA	NA	NA	NA
98	NA	Madagascar	NA	NA	NA	NA	NA
98	NA	Malawi	NA	NA	NA	NA	NA
98	NA	Maldives	NA	NA	NA	NA	NA
98	NA	Marshall Islands	NA	NA	NA	NA	NA
98	NA	Mauritania	NA	NA	NA	NA	NA
98	NA	Micronesia	NA	NA	NA	NA	NA
98	NA	Monaco	NA	NA	NA	NA	NA
98	NA	Mongolia	NA	NA	NA	NA	NA
98	NA	Montenegro	NA	NA	NA	NA	NA

98	NA	Namibia	NA	NA	NA	NA	NA
98	NA	Nauru	NA	NA	NA	NA	NA
98	NA	Nepal	NA	NA	NA	NA	NA
98	NA	New Zealand	NA	NA	NA	NA	NA
98	NA	Nicaragua	NA	NA	NA	NA	NA
98	NA	Niger	NA	NA	NA	NA	NA
98	NA	Niue	NA	NA	NA	NA	NA
98	NA	North Macedonia	NA	NA	NA	NA	NA
98	NA	Norway	NA	NA	NA	NA	NA
98	NA	Oman	NA	NA	NA	NA	NA
98	NA	Palau	NA	NA	NA	NA	NA
98	NA	Palestine	NA	NA	NA	NA	NA
98	NA	Papua New Guinea	NA	NA	NA	NA	NA
98	NA	Paraguay	NA	NA	NA	NA	NA
98	NA	Portugal	NA	NA	NA	NA	NA
98	NA	Qatar	NA	NA	NA	NA	NA
98	NA	Rwanda	NA	NA	NA	NA	NA
98	NA	Saint Kitts and Nevis	NA	NA	NA	NA	NA
98	NA	Saint Lucia	NA	NA	NA	NA	NA
98	NA	Saint Vincent and the Grenadines	NA	NA	NA	NA	NA
98	NA	Samoa	NA	NA	NA	NA	NA
98	NA	San Marino	NA	NA	NA	NA	NA
98	NA	Sao Tome and Principe	NA	NA	NA	NA	NA
98	NA	Saudi Arabia	NA	NA	NA	NA	NA
98	NA	Singapore	NA	NA	NA	NA	NA
98	NA	Slovakia	NA	NA	NA	NA	NA
98	NA	Solomon Islands	NA	NA	NA	NA	NA
98	NA	Somalia	NA	NA	NA	NA	NA
98	NA	South Sudan	NA	NA	NA	NA	NA
98	NA	Sri Lanka	NA	NA	NA	NA	NA
98	NA	Sudan	NA	NA	NA	NA	NA
98	NA	Suriname	NA	NA	NA	NA	NA
98	NA	Tajikistan	NA	NA	NA	NA	NA
98	NA	Tanzania	NA	NA	NA	NA	NA
98	NA	Tonga	NA	NA	NA	NA	NA
98	NA	Trinidad and Tobago	NA	NA	NA	NA	NA
98	NA	Turkmenistan	NA	NA	NA	NA	NA
98	NA	Tuvalu	NA	NA	NA	NA	NA
98	NA	Uruguay	NA	NA	NA	NA	NA
98	NA	Uzbekistan	NA	NA	NA	NA	NA
98	NA	Vanuatu	NA	NA	NA	NA	NA
98	NA	Vatican City	NA	NA	NA	NA	NA
98	NA	Yemen	NA	NA	NA	NA	NA
98	NA	Zimbabwe	NA	NA	NA	NA	NA

Table S2. World Cybercrime Index - Technical products/services

Rank	Noms	Country	I	P	TS	Overall	WCI Score
1	84	Russia	9.08	8.99	8.93	9.00	82.17
2	59	Ukraine	8.17	8.34	8.27	8.26	52.97
3	46	China	8.26	7.70	8.17	8.04	40.22
4	34	USA	7.68	7.06	7.71	7.48	27.64
5	23	Romania	7.26	6.83	7.30	7.13	17.83
6	20	Brazil	6.45	6.20	6.25	6.30	13.70
7	16	Belarus	6.44	6.94	7.19	6.85	11.92
8	10	North Korea	8.30	7.80	7.80	7.97	8.66
9	11	Iran	7.55	6.91	7.18	7.21	8.62
10	12	Nigeria	7.00	5.83	5.42	6.08	7.93
11	9	Moldova	6.89	6.56	7.11	6.85	6.70
12	7	Israel	8.14	8.14	8.43	8.24	6.27
13	6	UK	7.83	7.50	7.83	7.72	5.04
14	6	India	7.00	6.67	6.83	6.83	4.46
15	4	Germany	8.00	7.50	7.50	7.67	3.33
16	3	Estonia	8.33	6.67	7.67	7.56	2.46
17	3	Vietnam	6.33	5.67	6.33	6.11	1.99
18	2	Netherlands	8.00	8.00	9.00	8.33	1.81
19	3	France	5.67	5.00	5.67	5.44	1.78
20	2	South Korea	7.50	7.50	6.50	7.17	1.56
21	2	Latvia	6.00	7.50	7.00	6.83	1.49
22	2	Kazakhstan	7.00	6.50	6.50	6.67	1.45
23	2	Bulgaria	6.50	5.50	5.50	5.83	1.27
24	2	Ghana	6.50	4.00	6.50	5.67	1.23
25	2	South Africa	7.50	3.00	6.00	5.50	1.20
26	1	Poland	10.00	10.00	10.00	10.00	1.09
27	1	Cameroon	9.00	9.00	7.00	8.33	0.91
28	1	Czech Republic	8.00	9.00	7.00	8.00	0.87
28	1	Mauritius	9.00	7.00	8.00	8.00	0.87
30	1	Gambia	10.00	10.00	3.00	7.67	0.83
31	1	Lebanon	8.00	8.00	6.00	7.33	0.80
32	1	Italy	7.00	7.00	7.00	7.00	0.76
32	1	Hungary	7.00	7.00	7.00	7.00	0.76
34	1	Pakistan	7.00	6.00	6.00	6.33	0.69
34	1	Georgia	6.00	7.00	7.00	6.33	0.69
34	1	Bosnia and Herzegovina	5.00	7.00	7.00	6.33	0.69
37	1	Malaysia	6.00	5.00	6.00	5.67	0.62
38	1	Senegal	9.00	3.00	4.00	5.33	0.58
39	1	Turkey	5.00	5.00	5.00	5.00	0.54
39	1	Australia	7.00	4.00	4.00	5.00	0.54
41	1	Panama	3.00	5.00	5.00	4.33	0.47
41	1	Mali	10.00	1.00	2.00	4.33	0.47
43	1	Sierra Leone	10.00	1.00	1.00	4.00	0.43
43	1	Guinea-Bissau	10.00	1.00	1.00	4.00	0.43
45	NA	Afghanistan	NA	NA	NA	NA	NA
45	NA	Albania	NA	NA	NA	NA	NA
45	NA	Algeria	NA	NA	NA	NA	NA
45	NA	Andorra	NA	NA	NA	NA	NA
45	NA	Angola	NA	NA	NA	NA	NA

45	NA	Antigua and Barbuda	NA	NA	NA	NA	NA
45	NA	Argentina	NA	NA	NA	NA	NA
45	NA	Armenia	NA	NA	NA	NA	NA
45	NA	Austria	NA	NA	NA	NA	NA
45	NA	Azerbaijan	NA	NA	NA	NA	NA
45	NA	The Bahamas	NA	NA	NA	NA	NA
45	NA	Bahrain	NA	NA	NA	NA	NA
45	NA	Bangladesh	NA	NA	NA	NA	NA
45	NA	Barbados	NA	NA	NA	NA	NA
45	NA	Belgium	NA	NA	NA	NA	NA
45	NA	Belize	NA	NA	NA	NA	NA
45	NA	Benin	NA	NA	NA	NA	NA
45	NA	Bhutan	NA	NA	NA	NA	NA
45	NA	Bolivia	NA	NA	NA	NA	NA
45	NA	Botswana	NA	NA	NA	NA	NA
45	NA	Brunei	NA	NA	NA	NA	NA
45	NA	Burkina Faso	NA	NA	NA	NA	NA
45	NA	Burundi	NA	NA	NA	NA	NA
45	NA	Cambodia	NA	NA	NA	NA	NA
45	NA	Canada	NA	NA	NA	NA	NA
45	NA	Cape Verde	NA	NA	NA	NA	NA
45	NA	Central African Republic	NA	NA	NA	NA	NA
45	NA	Chad	NA	NA	NA	NA	NA
45	NA	Chile	NA	NA	NA	NA	NA
45	NA	Colombia	NA	NA	NA	NA	NA
45	NA	Comoros	NA	NA	NA	NA	NA
45	NA	Congo, Dem. Republic	NA	NA	NA	NA	NA
45	NA	Congo, Republic	NA	NA	NA	NA	NA
45	NA	Cook Islands	NA	NA	NA	NA	NA
45	NA	Costa Rica	NA	NA	NA	NA	NA
45	NA	Croatia	NA	NA	NA	NA	NA
45	NA	Cuba	NA	NA	NA	NA	NA
45	NA	Cyprus	NA	NA	NA	NA	NA
45	NA	Denmark	NA	NA	NA	NA	NA
45	NA	Djibouti	NA	NA	NA	NA	NA
45	NA	Dominica	NA	NA	NA	NA	NA
45	NA	Dominican Republic	NA	NA	NA	NA	NA
45	NA	East Timor	NA	NA	NA	NA	NA
45	NA	Ecuador	NA	NA	NA	NA	NA
45	NA	Egypt	NA	NA	NA	NA	NA
45	NA	El Salvador	NA	NA	NA	NA	NA
45	NA	Equatorial Guinea	NA	NA	NA	NA	NA
45	NA	Eritrea	NA	NA	NA	NA	NA
45	NA	Eswatini	NA	NA	NA	NA	NA
45	NA	Ethiopia	NA	NA	NA	NA	NA
45	NA	Fiji	NA	NA	NA	NA	NA
45	NA	Finland	NA	NA	NA	NA	NA
45	NA	Gabon	NA	NA	NA	NA	NA
45	NA	Greece	NA	NA	NA	NA	NA
45	NA	Grenada	NA	NA	NA	NA	NA
45	NA	Guatemala	NA	NA	NA	NA	NA
45	NA	Guinea	NA	NA	NA	NA	NA

45	NA	Guyana	NA	NA	NA	NA	NA
45	NA	Haiti	NA	NA	NA	NA	NA
45	NA	Honduras	NA	NA	NA	NA	NA
45	NA	Iceland	NA	NA	NA	NA	NA
45	NA	Indonesia	NA	NA	NA	NA	NA
45	NA	Iraq	NA	NA	NA	NA	NA
45	NA	Ireland	NA	NA	NA	NA	NA
45	NA	Ivory Coast	NA	NA	NA	NA	NA
45	NA	Jamaica	NA	NA	NA	NA	NA
45	NA	Japan	NA	NA	NA	NA	NA
45	NA	Jordan	NA	NA	NA	NA	NA
45	NA	Kenya	NA	NA	NA	NA	NA
45	NA	Kiribati	NA	NA	NA	NA	NA
45	NA	Kuwait	NA	NA	NA	NA	NA
45	NA	Kyrgyzstan	NA	NA	NA	NA	NA
45	NA	Laos	NA	NA	NA	NA	NA
45	NA	Lesotho	NA	NA	NA	NA	NA
45	NA	Liberia	NA	NA	NA	NA	NA
45	NA	Libya	NA	NA	NA	NA	NA
45	NA	Liechtenstein	NA	NA	NA	NA	NA
45	NA	Lithuania	NA	NA	NA	NA	NA
45	NA	Luxembourg	NA	NA	NA	NA	NA
45	NA	Madagascar	NA	NA	NA	NA	NA
45	NA	Malawi	NA	NA	NA	NA	NA
45	NA	Maldives	NA	NA	NA	NA	NA
45	NA	Malta	NA	NA	NA	NA	NA
45	NA	Marshall Islands	NA	NA	NA	NA	NA
45	NA	Mauritania	NA	NA	NA	NA	NA
45	NA	Mexico	NA	NA	NA	NA	NA
45	NA	Micronesia	NA	NA	NA	NA	NA
45	NA	Monaco	NA	NA	NA	NA	NA
45	NA	Mongolia	NA	NA	NA	NA	NA
45	NA	Montenegro	NA	NA	NA	NA	NA
45	NA	Morocco	NA	NA	NA	NA	NA
45	NA	Mozambique	NA	NA	NA	NA	NA
45	NA	Myanmar	NA	NA	NA	NA	NA
45	NA	Namibia	NA	NA	NA	NA	NA
45	NA	Nauru	NA	NA	NA	NA	NA
45	NA	Nepal	NA	NA	NA	NA	NA
45	NA	New Zealand	NA	NA	NA	NA	NA
45	NA	Nicaragua	NA	NA	NA	NA	NA
45	NA	Niger	NA	NA	NA	NA	NA
45	NA	Niue	NA	NA	NA	NA	NA
45	NA	North Macedonia	NA	NA	NA	NA	NA
45	NA	Norway	NA	NA	NA	NA	NA
45	NA	Oman	NA	NA	NA	NA	NA
45	NA	Palau	NA	NA	NA	NA	NA
45	NA	Palestine	NA	NA	NA	NA	NA
45	NA	Papua New Guinea	NA	NA	NA	NA	NA
45	NA	Paraguay	NA	NA	NA	NA	NA
45	NA	Peru	NA	NA	NA	NA	NA
45	NA	Philippines	NA	NA	NA	NA	NA

45	NA	Portugal	NA	NA	NA	NA	NA
45	NA	Qatar	NA	NA	NA	NA	NA
45	NA	Rwanda	NA	NA	NA	NA	NA
45	NA	Saint Kitts and Nevis	NA	NA	NA	NA	NA
45	NA	Saint Lucia	NA	NA	NA	NA	NA
45	NA	Saint Vincent and the Grenadines	NA	NA	NA	NA	NA
45	NA	Samoa	NA	NA	NA	NA	NA
45	NA	San Marino	NA	NA	NA	NA	NA
45	NA	Sao Tome and Principe	NA	NA	NA	NA	NA
45	NA	Saudi Arabia	NA	NA	NA	NA	NA
45	NA	Serbia	NA	NA	NA	NA	NA
45	NA	Seychelles	NA	NA	NA	NA	NA
45	NA	Singapore	NA	NA	NA	NA	NA
45	NA	Slovakia	NA	NA	NA	NA	NA
45	NA	Slovenia	NA	NA	NA	NA	NA
45	NA	Solomon Islands	NA	NA	NA	NA	NA
45	NA	Somalia	NA	NA	NA	NA	NA
45	NA	South Sudan	NA	NA	NA	NA	NA
45	NA	Spain	NA	NA	NA	NA	NA
45	NA	Sri Lanka	NA	NA	NA	NA	NA
45	NA	Sudan	NA	NA	NA	NA	NA
45	NA	Suriname	NA	NA	NA	NA	NA
45	NA	Sweden	NA	NA	NA	NA	NA
45	NA	Switzerland	NA	NA	NA	NA	NA
45	NA	Syria	NA	NA	NA	NA	NA
45	NA	Tajikistan	NA	NA	NA	NA	NA
45	NA	Tanzania	NA	NA	NA	NA	NA
45	NA	Thailand	NA	NA	NA	NA	NA
45	NA	Togo	NA	NA	NA	NA	NA
45	NA	Tonga	NA	NA	NA	NA	NA
45	NA	Trinidad and Tobago	NA	NA	NA	NA	NA
45	NA	Tunisia	NA	NA	NA	NA	NA
45	NA	Turkmenistan	NA	NA	NA	NA	NA
45	NA	Tuvalu	NA	NA	NA	NA	NA
45	NA	Uganda	NA	NA	NA	NA	NA
45	NA	United Arab Emirates	NA	NA	NA	NA	NA
45	NA	Uruguay	NA	NA	NA	NA	NA
45	NA	Uzbekistan	NA	NA	NA	NA	NA
45	NA	Vanuatu	NA	NA	NA	NA	NA
45	NA	Vatican City	NA	NA	NA	NA	NA
45	NA	Venezuela	NA	NA	NA	NA	NA
45	NA	Yemen	NA	NA	NA	NA	NA
45	NA	Zambia	NA	NA	NA	NA	NA
45	NA	Zimbabwe	NA	NA	NA	NA	NA

Table S3. World Cybercrime Index - Attacks and extortion

Rank	Noms	Country	I	P	TS	Overall	WCI Score
1	83	Russia	9.29	8.93	8.83	9.02	81.34
2	56	Ukraine	8.46	8.20	8.36	8.34	50.76
3	31	North Korea	8.10	7.10	7.35	7.52	25.33
4	28	China	8.18	7.82	7.89	7.96	24.24
5	23	USA	7.43	6.78	7.00	7.07	17.68
6	14	Iran	6.79	6.43	6.50	6.57	10.00
7	13	Romania	6.31	6.31	6.85	6.49	9.17
8	12	Brazil	7.25	6.25	6.67	6.72	8.77
9	11	Nigeria	7.55	6.73	6.82	7.03	8.41
10	7	Belarus	7.29	7.43	7.29	7.33	5.58
11	7	UK	7.00	5.71	6.00	6.24	4.75
12	5	Kazakhstan	7.00	6.80	6.80	6.87	3.73
13	5	India	7.60	6.00	6.40	6.67	3.62
14	3	Netherlands	8.00	7.33	7.67	7.67	2.50
15	2	Israel	9.50	9.50	10.00	9.67	2.10
16	4	Turkey	5.00	4.50	4.75	4.75	2.07
17	2	Poland	9.00	8.50	8.50	8.67	1.88
18	2	France	8.00	8.50	8.00	8.17	1.78
19	2	Latvia	6.50	7.50	8.00	7.33	1.59
20	2	Estonia	3.50	7.50	7.50	6.17	1.34
20	2	Bulgaria	6.50	5.50	6.50	6.17	1.34
22	1	Sweden	9.00	10.00	10.00	9.67	1.05
23	1	Moldova	9.00	9.00	9.00	9.00	0.98
24	1	Lithuania	9.00	8.00	9.00	8.67	0.94
25	1	Germany	9.00	8.00	8.00	8.33	0.91
25	1	Belgium	9.00	8.00	8.00	8.33	0.91
27	1	Canada	10.00	6.00	8.00	8.00	0.87
28	1	Ghana	5.00	7.00	9.00	7.00	0.76
28	1	South Korea	7.00	7.00	7.00	7.00	0.76
28	1	Czech Republic	7.00	7.00	7.00	7.00	0.76
28	1	Benin	6.00	7.00	8.00	7.00	0.76
28	1	Slovenia	7.00	7.00	7.00	7.00	0.76
33	1	Malaysia	8.00	6.00	6.00	6.67	0.72
34	1	Vietnam	8.00	5.00	6.00	6.33	0.69
34	1	Angola	7.00	3.00	9.00	6.33	0.69
36	1	South Africa	6.00	4.00	8.00	6.00	0.65
36	1	Indonesia	6.00	6.00	6.00	6.00	0.65
36	1	Georgia	6.00	6.00	6.00	6.00	0.65
39	1	Mexico	7.00	4.00	4.00	5.00	0.54
39	1	Japan	5.00	4.00	6.00	5.00	0.54
41	1	Pakistan	6.00	3.00	4.00	4.33	0.47
41	1	Syria	3.00	5.00	5.00	4.33	0.47
43	1	Philippines	5.00	3.00	3.00	3.67	0.40
43	1	Switzerland	3.00	4.00	4.00	3.67	0.40
43	1	Egypt	3.00	3.00	5.00	3.67	0.40
46	NA	Afghanistan	NA	NA	NA	NA	NA
46	NA	Albania	NA	NA	NA	NA	NA
46	NA	Algeria	NA	NA	NA	NA	NA
46	NA	Andorra	NA	NA	NA	NA	NA

46	NA	Angola	NA	NA	NA	NA	NA
46	NA	Antigua and Barbuda	NA	NA	NA	NA	NA
46	NA	Argentina	NA	NA	NA	NA	NA
46	NA	Armenia	NA	NA	NA	NA	NA
46	NA	Australia	NA	NA	NA	NA	NA
46	NA	Austria	NA	NA	NA	NA	NA
46	NA	Azerbaijan	NA	NA	NA	NA	NA
46	NA	The Bahamas	NA	NA	NA	NA	NA
46	NA	Bahrain	NA	NA	NA	NA	NA
46	NA	Bangladesh	NA	NA	NA	NA	NA
46	NA	Barbados	NA	NA	NA	NA	NA
46	NA	Belize	NA	NA	NA	NA	NA
46	NA	Bhutan	NA	NA	NA	NA	NA
46	NA	Bolivia	NA	NA	NA	NA	NA
46	NA	Bosnia and Herzegovina	NA	NA	NA	NA	NA
46	NA	Botswana	NA	NA	NA	NA	NA
46	NA	Brunei	NA	NA	NA	NA	NA
46	NA	Burkina Faso	NA	NA	NA	NA	NA
46	NA	Burundi	NA	NA	NA	NA	NA
46	NA	Cambodia	NA	NA	NA	NA	NA
46	NA	Cameroon	NA	NA	NA	NA	NA
46	NA	Cape Verde	NA	NA	NA	NA	NA
46	NA	Central African Republic	NA	NA	NA	NA	NA
46	NA	Chad	NA	NA	NA	NA	NA
46	NA	Chile	NA	NA	NA	NA	NA
46	NA	Colombia	NA	NA	NA	NA	NA
46	NA	Comoros	NA	NA	NA	NA	NA
46	NA	Congo, Dem. Republic	NA	NA	NA	NA	NA
46	NA	Congo, Republic	NA	NA	NA	NA	NA
46	NA	Cook Islands	NA	NA	NA	NA	NA
46	NA	Costa Rica	NA	NA	NA	NA	NA
46	NA	Croatia	NA	NA	NA	NA	NA
46	NA	Cuba	NA	NA	NA	NA	NA
46	NA	Cyprus	NA	NA	NA	NA	NA
46	NA	Denmark	NA	NA	NA	NA	NA
46	NA	Djibouti	NA	NA	NA	NA	NA
46	NA	Dominica	NA	NA	NA	NA	NA
46	NA	Dominican Republic	NA	NA	NA	NA	NA
46	NA	East Timor	NA	NA	NA	NA	NA
46	NA	Ecuador	NA	NA	NA	NA	NA
46	NA	El Salvador	NA	NA	NA	NA	NA
46	NA	Equatorial Guinea	NA	NA	NA	NA	NA
46	NA	Eritrea	NA	NA	NA	NA	NA
46	NA	Eswatini	NA	NA	NA	NA	NA
46	NA	Ethiopia	NA	NA	NA	NA	NA
46	NA	Fiji	NA	NA	NA	NA	NA
46	NA	Finland	NA	NA	NA	NA	NA
46	NA	Gabon	NA	NA	NA	NA	NA
46	NA	Gambia	NA	NA	NA	NA	NA
46	NA	Greece	NA	NA	NA	NA	NA
46	NA	Grenada	NA	NA	NA	NA	NA
46	NA	Guatemala	NA	NA	NA	NA	NA

46	NA	Guinea	NA	NA	NA	NA	NA
46	NA	Guinea-Bissau	NA	NA	NA	NA	NA
46	NA	Guyana	NA	NA	NA	NA	NA
46	NA	Haiti	NA	NA	NA	NA	NA
46	NA	Honduras	NA	NA	NA	NA	NA
46	NA	Hungary	NA	NA	NA	NA	NA
46	NA	Iceland	NA	NA	NA	NA	NA
46	NA	Iraq	NA	NA	NA	NA	NA
46	NA	Ireland	NA	NA	NA	NA	NA
46	NA	Italy	NA	NA	NA	NA	NA
46	NA	Ivory Coast	NA	NA	NA	NA	NA
46	NA	Jamaica	NA	NA	NA	NA	NA
46	NA	Jordan	NA	NA	NA	NA	NA
46	NA	Kenya	NA	NA	NA	NA	NA
46	NA	Kiribati	NA	NA	NA	NA	NA
46	NA	Kuwait	NA	NA	NA	NA	NA
46	NA	Kyrgyzstan	NA	NA	NA	NA	NA
46	NA	Laos	NA	NA	NA	NA	NA
46	NA	Lebanon	NA	NA	NA	NA	NA
46	NA	Lesotho	NA	NA	NA	NA	NA
46	NA	Liberia	NA	NA	NA	NA	NA
46	NA	Libya	NA	NA	NA	NA	NA
46	NA	Liechtenstein	NA	NA	NA	NA	NA
46	NA	Luxembourg	NA	NA	NA	NA	NA
46	NA	Madagascar	NA	NA	NA	NA	NA
46	NA	Malawi	NA	NA	NA	NA	NA
46	NA	Maldives	NA	NA	NA	NA	NA
46	NA	Mali	NA	NA	NA	NA	NA
46	NA	Malta	NA	NA	NA	NA	NA
46	NA	Marshall Islands	NA	NA	NA	NA	NA
46	NA	Mauritania	NA	NA	NA	NA	NA
46	NA	Mauritius	NA	NA	NA	NA	NA
46	NA	Micronesia	NA	NA	NA	NA	NA
46	NA	Monaco	NA	NA	NA	NA	NA
46	NA	Mongolia	NA	NA	NA	NA	NA
46	NA	Montenegro	NA	NA	NA	NA	NA
46	NA	Morocco	NA	NA	NA	NA	NA
46	NA	Mozambique	NA	NA	NA	NA	NA
46	NA	Myanmar	NA	NA	NA	NA	NA
46	NA	Namibia	NA	NA	NA	NA	NA
46	NA	Nauru	NA	NA	NA	NA	NA
46	NA	Nepal	NA	NA	NA	NA	NA
46	NA	New Zealand	NA	NA	NA	NA	NA
46	NA	Nicaragua	NA	NA	NA	NA	NA
46	NA	Niger	NA	NA	NA	NA	NA
46	NA	Niue	NA	NA	NA	NA	NA
46	NA	North Macedonia	NA	NA	NA	NA	NA
46	NA	Norway	NA	NA	NA	NA	NA
46	NA	Oman	NA	NA	NA	NA	NA
46	NA	Palau	NA	NA	NA	NA	NA
46	NA	Palestine	NA	NA	NA	NA	NA
46	NA	Panama	NA	NA	NA	NA	NA

46	NA	Papua New Guinea	NA	NA	NA	NA	NA
46	NA	Paraguay	NA	NA	NA	NA	NA
46	NA	Peru	NA	NA	NA	NA	NA
46	NA	Portugal	NA	NA	NA	NA	NA
46	NA	Qatar	NA	NA	NA	NA	NA
46	NA	Rwanda	NA	NA	NA	NA	NA
46	NA	Saint Kitts and Nevis	NA	NA	NA	NA	NA
46	NA	Saint Lucia	NA	NA	NA	NA	NA
46	NA	Saint Vincent and the Grenadines	NA	NA	NA	NA	NA
46	NA	Samoa	NA	NA	NA	NA	NA
46	NA	San Marino	NA	NA	NA	NA	NA
46	NA	Sao Tome and Principe	NA	NA	NA	NA	NA
46	NA	Saudi Arabia	NA	NA	NA	NA	NA
46	NA	Senegal	NA	NA	NA	NA	NA
46	NA	Serbia	NA	NA	NA	NA	NA
46	NA	Seychelles	NA	NA	NA	NA	NA
46	NA	Sierra Leone	NA	NA	NA	NA	NA
46	NA	Singapore	NA	NA	NA	NA	NA
46	NA	Slovakia	NA	NA	NA	NA	NA
46	NA	Solomon Islands	NA	NA	NA	NA	NA
46	NA	Somalia	NA	NA	NA	NA	NA
46	NA	South Sudan	NA	NA	NA	NA	NA
46	NA	Spain	NA	NA	NA	NA	NA
46	NA	Sri Lanka	NA	NA	NA	NA	NA
46	NA	Sudan	NA	NA	NA	NA	NA
46	NA	Suriname	NA	NA	NA	NA	NA
46	NA	Tajikistan	NA	NA	NA	NA	NA
46	NA	Tanzania	NA	NA	NA	NA	NA
46	NA	Thailand	NA	NA	NA	NA	NA
46	NA	Togo	NA	NA	NA	NA	NA
46	NA	Tonga	NA	NA	NA	NA	NA
46	NA	Trinidad and Tobago	NA	NA	NA	NA	NA
46	NA	Tunisia	NA	NA	NA	NA	NA
46	NA	Turkmenistan	NA	NA	NA	NA	NA
46	NA	Tuvalu	NA	NA	NA	NA	NA
46	NA	Uganda	NA	NA	NA	NA	NA
46	NA	United Arab Emirates	NA	NA	NA	NA	NA
46	NA	Uruguay	NA	NA	NA	NA	NA
46	NA	Uzbekistan	NA	NA	NA	NA	NA
46	NA	Vanuatu	NA	NA	NA	NA	NA
46	NA	Vatican City	NA	NA	NA	NA	NA
46	NA	Venezuela	NA	NA	NA	NA	NA
46	NA	Yemen	NA	NA	NA	NA	NA
46	NA	Zambia	NA	NA	NA	NA	NA
46	NA	Zimbabwe	NA	NA	NA	NA	NA

11

Table S4. World Cybercrime Index - Data/identity theft

Rank	Noms	Country	I	P	TS	Overall	WCI Score
1	67	Russia	9.09	8.90	87	8.95	65.18
2	39	Ukraine	8.59	8.51	8.38	8.50	36.01
3	40	China	8.22	7.70	8.15	8.03	34.89
4	36	USA	8.08	7.53	7.67	7.76	30.36
5	31	Nigeria	8.58	6.10	5.84	6.84	23.04
6	28	Romania	7.18	7.57	7.43	7.39	22.50
7	16	North Korea	7.62	7.31	7.50	7.48	13.01
8	14	Brazil	7.21	6.64	6.43	6.76	10.29
9	9	India	8.11	6.44	6.33	6.96	6.81
10	7	UK	8.14	7.43	7.29	7.62	5.80
11	6	Iran	5.67	5.33	5.50	5.50	3.59
12	4	Ghana	9.00	6.00	5.50	6.83	2.97
13	3	Latvia	8.00	8.67	8.33	8.33	2.72
14	4	Vietnam	7.50	6.25	4.75	6.17	2.68
15	3	Moldova	7.33	7.33	7.67	7.44	2.43
16	3	Thailand	7.67	7.00	6.67	7.11	2.32
17	3	Kazakhstan	7.00	7.00	7.33	7.11	2.32
18	3	Indonesia	7.33	7.33	6.33	7.00	2.28
19	3	Netherlands	7.33	7.00	6.33	6.89	2.25
20	3	Germany	6.67	6.67	6.33	6.56	2.14
21	2	Belarus	8.50	8.50	8.50	8.50	1.85
21	2	Israel	8.50	8.50	8.50	8.50	1.85
23	2	Canada	8.50	8.00	8.00	8.17	1.78
24	3	Morocco	6.67	4.67	4.33	5.22	1.70
24	3	Algeria	6.67	4.67	4.33	5.22	1.70
26	2	Poland	8.00	8.00	7.00	7.67	1.67
27	2	Australia	7.00	7.50	7.50	7.33	1.59
28	2	Bulgaria	5.50	7.00	7.00	6.50	1.41
29	2	Malaysia	6.50	5.00	5.50	5.67	1.23
30	2	Philippines	7.00	4.50	4.00	5.17	1.12
30	2	Tunisia	7.00	4.50	4.00	5.17	1.12
32	1	Armenia	9.00	9.00	9.00	9.00	0.98
32	1	Lithuania	9.00	9.00	9.00	9.00	0.98
32	1	Cuba	9.00	9.00	9.00	9.00	9.00
35	1	Uganda	10.00	7.00	9.00	8.67	0.94
36	1	France	8.00	8.00	8.00	8.00	0.87
36	1	Lebanon	8.00	8.00	8.00	8.00	0.87
38	1	Kenya	10.00	7.00	6.00	7.67	0.83
39	1	Estonia	5.00	8.00	8.00	7.00	0.76
39	1	South Korea	7.00	7.00	7.00	7.00	0.76
39	1	Cambodia	10.00	10.00	1.00	7.00	0.76
39	1	Myanmar	10.00	10.00	1.00	7.00	0.76
39	1	Serbia	7.00	7.00	7.00	7.00	0.76
44	1	Gambia	10.00	2.00	8.00	6.67	0.72
45	1	Colombia	7.00	6.00	6.00	6.33	0.69
45	1	Chile	7.00	6.00	6.00	6.33	0.69
45	1	Laos	9.00	9.00	1.00	6.33	0.69
45	1	Senegal	10.00	4.00	5.00	6.33	0.69
49	1	Pakistan	7.00	5.00	5.00	5.67	0.62

49	1	Ecuador	6.00	6.00	5.00	5.67	0.62
49	1	Peru	6.00	6.00	5.00	5.67	0.62
52	1	South Africa	6.00	5.00	5.00	5.33	0.58
53	1	Mexico	4.00	4.00	3.00	3.67	0.40
53	1	Ivory Coast	3.00	4.00	4.00	3.67	0.40
55	NA	Afghanistan	NA	NA	NA	NA	NA
55	NA	Albania	NA	NA	NA	NA	NA
55	NA	Andorra	NA	NA	NA	NA	NA
55	NA	Angola	NA	NA	NA	NA	NA
55	NA	Antigua and Barbuda	NA	NA	NA	NA	NA
55	NA	Argentina	NA	NA	NA	NA	NA
55	NA	Austria	NA	NA	NA	NA	NA
55	NA	Azerbaijan	NA	NA	NA	NA	NA
55	NA	The Bahamas	NA	NA	NA	NA	NA
55	NA	Bahrain	NA	NA	NA	NA	NA
55	NA	Bangladesh	NA	NA	NA	NA	NA
55	NA	Barbados	NA	NA	NA	NA	NA
55	NA	Belgium	NA	NA	NA	NA	NA
55	NA	Belize	NA	NA	NA	NA	NA
55	NA	Benin	NA	NA	NA	NA	NA
55	NA	Bhutan	NA	NA	NA	NA	NA
55	NA	Bolivia	NA	NA	NA	NA	NA
55	NA	Bosnia and Herzegovina	NA	NA	NA	NA	NA
55	NA	Botswana	NA	NA	NA	NA	NA
55	NA	Brunei	NA	NA	NA	NA	NA
55	NA	Burkina Faso	NA	NA	NA	NA	NA
55	NA	Burundi	NA	NA	NA	NA	NA
55	NA	Cambodia	NA	NA	NA	NA	NA
55	NA	Cameroon	NA	NA	NA	NA	NA
55	NA	Cape Verde	NA	NA	NA	NA	NA
55	NA	Central African Republic	NA	NA	NA	NA	NA
55	NA	Chad	NA	NA	NA	NA	NA
55	NA	Comoros	NA	NA	NA	NA	NA
55	NA	Congo, Dem. Republic	NA	NA	NA	NA	NA
55	NA	Congo, Republic	NA	NA	NA	NA	NA
55	NA	Cook Islands	NA	NA	NA	NA	NA
55	NA	Costa Rica	NA	NA	NA	NA	NA
55	NA	Croatia	NA	NA	NA	NA	NA
55	NA	Cyprus	NA	NA	NA	NA	NA
55	NA	Czech Republic	NA	NA	NA	NA	NA
55	NA	Denmark	NA	NA	NA	NA	NA
55	NA	Djibouti	NA	NA	NA	NA	NA
55	NA	Dominica	NA	NA	NA	NA	NA
55	NA	Dominican Republic	NA	NA	NA	NA	NA
55	NA	East Timor	NA	NA	NA	NA	NA
55	NA	Egypt	NA	NA	NA	NA	NA
55	NA	El Salvador	NA	NA	NA	NA	NA
55	NA	Equatorial Guinea	NA	NA	NA	NA	NA
55	NA	Eritrea	NA	NA	NA	NA	NA
55	NA	Eswatini	NA	NA	NA	NA	NA
55	NA	Ethiopia	NA	NA	NA	NA	NA
55	NA	Fiji	NA	NA	NA	NA	NA

55	NA	Finland	NA	NA	NA	NA	NA
55	NA	Gabon	NA	NA	NA	NA	NA
55	NA	Georgia	NA	NA	NA	NA	NA
55	NA	Greece	NA	NA	NA	NA	NA
55	NA	Grenada	NA	NA	NA	NA	NA
55	NA	Guatemala	NA	NA	NA	NA	NA
55	NA	Guinea	NA	NA	NA	NA	NA
55	NA	Guinea-Bissau	NA	NA	NA	NA	NA
55	NA	Guyana	NA	NA	NA	NA	NA
55	NA	Haiti	NA	NA	NA	NA	NA
55	NA	Honduras	NA	NA	NA	NA	NA
55	NA	Hungary	NA	NA	NA	NA	NA
55	NA	Iceland	NA	NA	NA	NA	NA
55	NA	Iraq	NA	NA	NA	NA	NA
55	NA	Ireland	NA	NA	NA	NA	NA
55	NA	Italy	NA	NA	NA	NA	NA
55	NA	Jamaica	NA	NA	NA	NA	NA
55	NA	Japan	NA	NA	NA	NA	NA
55	NA	Jordan	NA	NA	NA	NA	NA
55	NA	Kiribati	NA	NA	NA	NA	NA
55	NA	Kuwait	NA	NA	NA	NA	NA
55	NA	Kyrgyzstan	NA	NA	NA	NA	NA
55	NA	Lesotho	NA	NA	NA	NA	NA
55	NA	Liberia	NA	NA	NA	NA	NA
55	NA	Libya	NA	NA	NA	NA	NA
55	NA	Liechtenstein	NA	NA	NA	NA	NA
55	NA	Luxembourg	NA	NA	NA	NA	NA
55	NA	Madagascar	NA	NA	NA	NA	NA
55	NA	Malawi	NA	NA	NA	NA	NA
55	NA	Maldives	NA	NA	NA	NA	NA
55	NA	Mali	NA	NA	NA	NA	NA
55	NA	Malta	NA	NA	NA	NA	NA
55	NA	Marshall Islands	NA	NA	NA	NA	NA
55	NA	Mauritania	NA	NA	NA	NA	NA
55	NA	Mauritius	NA	NA	NA	NA	NA
55	NA	Micronesia	NA	NA	NA	NA	NA
55	NA	Monaco	NA	NA	NA	NA	NA
55	NA	Mongolia	NA	NA	NA	NA	NA
55	NA	Montenegro	NA	NA	NA	NA	NA
55	NA	Mozambique	NA	NA	NA	NA	NA
55	NA	Myanmar	NA	NA	NA	NA	NA
55	NA	Namibia	NA	NA	NA	NA	NA
55	NA	Nauru	NA	NA	NA	NA	NA
55	NA	Nepal	NA	NA	NA	NA	NA
55	NA	New Zealand	NA	NA	NA	NA	NA
55	NA	Nicaragua	NA	NA	NA	NA	NA
55	NA	Niger	NA	NA	NA	NA	NA
55	NA	Niue	NA	NA	NA	NA	NA
55	NA	North Macedonia	NA	NA	NA	NA	NA
55	NA	Norway	NA	NA	NA	NA	NA
55	NA	Oman	NA	NA	NA	NA	NA
55	NA	Palau	NA	NA	NA	NA	NA

55	NA	Palestine	NA	NA	NA	NA	NA
55	NA	Panama	NA	NA	NA	NA	NA
55	NA	Papua New Guinea	NA	NA	NA	NA	NA
55	NA	Paraguay	NA	NA	NA	NA	NA
55	NA	Portugal	NA	NA	NA	NA	NA
55	NA	Qatar	NA	NA	NA	NA	NA
55	NA	Rwanda	NA	NA	NA	NA	NA
55	NA	Saint Kitts and Nevis	NA	NA	NA	NA	NA
55	NA	Saint Lucia	NA	NA	NA	NA	NA
55	NA	Saint Vincent and the Grenadines	NA	NA	NA	NA	NA
55	NA	Samoa	NA	NA	NA	NA	NA
55	NA	San Marino	NA	NA	NA	NA	NA
55	NA	Sao Tome and Principe	NA	NA	NA	NA	NA
55	NA	Saudi Arabia	NA	NA	NA	NA	NA
55	NA	Seychelles	NA	NA	NA	NA	NA
55	NA	Sierra Leone	NA	NA	NA	NA	NA
55	NA	Singapore	NA	NA	NA	NA	NA
55	NA	Slovakia	NA	NA	NA	NA	NA
55	NA	Slovenia	NA	NA	NA	NA	NA
55	NA	Solomon Islands	NA	NA	NA	NA	NA
55	NA	Somalia	NA	NA	NA	NA	NA
55	NA	South Sudan	NA	NA	NA	NA	NA
55	NA	Spain	NA	NA	NA	NA	NA
55	NA	Sri Lanka	NA	NA	NA	NA	NA
55	NA	Sudan	NA	NA	NA	NA	NA
55	NA	Suriname	NA	NA	NA	NA	NA
55	NA	Sweden	NA	NA	NA	NA	NA
55	NA	Switzerland	NA	NA	NA	NA	NA
55	NA	Syria	NA	NA	NA	NA	NA
55	NA	Tajikistan	NA	NA	NA	NA	NA
55	NA	Tanzania	NA	NA	NA	NA	NA
55	NA	Togo	NA	NA	NA	NA	NA
55	NA	Tonga	NA	NA	NA	NA	NA
55	NA	Trinidad and Tobago	NA	NA	NA	NA	NA
55	NA	Turkey	NA	NA	NA	NA	NA
55	NA	Turkmenistan	NA	NA	NA	NA	NA
55	NA	Tuvalu	NA	NA	NA	NA	NA
55	NA	United Arab Emirates	NA	NA	NA	NA	NA
55	NA	Uruguay	NA	NA	NA	NA	NA
55	NA	Uzbekistan	NA	NA	NA	NA	NA
55	NA	Vanuatu	NA	NA	NA	NA	NA
55	NA	Vatican City	NA	NA	NA	NA	NA
55	NA	Venezuela	NA	NA	NA	NA	NA
55	NA	Yemen	NA	NA	NA	NA	NA
55	NA	Zambia	NA	NA	NA	NA	NA
55	NA	Zimbabwe	NA	NA	NA	NA	NA

12

Table S5. World Cybercrime Index - Scams

Rank	Noms	Country	I	P	TS	Overall	WCI Score
1	70	Nigeria	8.37	6.57	5.63	6.86	52.17
2	27	USA	8.52	7.59	7.11	7.74	22.72
3	25	Russia	7.76	7.92	8.28	7.99	21.70
4	19	China	8.16	7.53	7.32	7.67	15.83
5	17	Romania	7.24	7.12	7.00	7.12	13.15
6	16	India	8.25	6.88	6.88	7.33	12.75
7	14	Ukraine	7.21	7.57	7.29	7.36	11.20
8	13	Ghana	8.69	7.08	6.23	7.33	10.36
9	10	UK	7.70	7.20	6.80	7.23	7.86
10	10	Brazil	7.10	6.50	6.50	6.70	7.28
11	11	South Africa	7.18	5.55	5.27	6.00	7.17
12	7	Malaysia	6.00	4.14	3.43	4.52	3.44
13	4	Indonesia	6.75	6.75	6.50	6.67	2.90
14	3	Canada	7.67	8.33	9.00	8.33	2.72
15	4	Cameroon	8.00	5.00	5.00	6.00	2.61
16	4	Philippines	7.25	5.50	4.25	5.67	2.46
17	3	Poland	8.00	7.67	6.67	7.44	2.43
18	3	Mexico	7.33	6.33	6.67	6.78	2.21
19	3	North Korea	6.33	6.67	7.00	6.67	2.17
20	3	UAE	7.33	5.33	5.67	6.11	1.99
21	3	Vietnam	7.00	6.00	4.67	5.89	1.92
22	2	Israel	8.00	7.50	7.00	7.50	1.63
22	2	Italy	7.50	7.50	7.50	7.50	1.63
24	2	Gambia	9.00	6.00	6.50	7.17	1.56
25	2	Netherlands	7.50	7.00	6.00	6.83	1.49
26	2	Sierra Leone	9.00	4.50	6.50	6.67	1.45
27	2	Colombia	7.00	6.50	5.00	6.17	1.34
28	3	Ivory Coast	6.00	4.00	2.33	4.11	1.34
29	2	Australia	7.00	5.00	5.00	5.67	1.23
30	1	Iran	8.00	9.00	9.00	8.67	0.94
30	1	Greece	10.00	8.00	8.00	8.67	0.94
32	1	South Korea	8.00	8.00	8.00	8.00	0.87
33	1	Moldova	7.00	8.00	8.00	7.67	0.83
34	1	France	7.00	7.00	8.00	7.33	0.80
34	1	Laos	10.00	10.00	2.00	7.33	0.80
36	1	Bulgaria	8.00	7.00	6.00	7.00	0.76
36	1	Pakistan	7.00	7.00	7.00	7.00	0.76
38	1	Jamaica	9.00	7.00	4.00	6.67	0.72
39	1	Latvia	6.00	7.00	6.00	6.33	0.69
39	1	Cambodia	9.00	9.00	1.00	6.33	0.69
39	1	Venezuela	4.00	3.00	2.50	3.17	0.69
39	1	Central African Republic	7.00	7.00	5.00	6.33	0.69
43	1	Dominican Republic	9.00	7.00	2.00	6.00	0.65
44	1	Thailand	8.00	7.00	1.00	5.33	0.58
44	1	Spain	6.00	5.00	5.00	5.33	0.58
46	1	Morocco	7.00	5.00	3.00	5.00	0.54
46	1	Chile	6.00	5.00	4.00	5.00	0.54
46	1	Ecuador	6.00	5.00	4.00	5.00	0.54
46	1	Peru	6.00	5.00	4.00	5.00	0.54

50	1	Botswana	3.00	3.00	3.00	3.00	0.33
50	1	Ethiopia	3.00	3.00	3.00	3.00	0.33
50	1	Zambia	3.00	3.00	3.00	3.00	0.33
55	NA	Afghanistan	NA	NA	NA	NA	NA
55	NA	Albania	NA	NA	NA	NA	NA
55	NA	Algeria	NA	NA	NA	NA	NA
55	NA	Andorra	NA	NA	NA	NA	NA
55	NA	Angola	NA	NA	NA	NA	NA
55	NA	Antigua and Barbuda	NA	NA	NA	NA	NA
55	NA	Argentina	NA	NA	NA	NA	NA
55	NA	Armenia	NA	NA	NA	NA	NA
55	NA	Austria	NA	NA	NA	NA	NA
55	NA	Azerbaijan	NA	NA	NA	NA	NA
55	NA	The Bahamas	NA	NA	NA	NA	NA
55	NA	Bahrain	NA	NA	NA	NA	NA
55	NA	Bangladesh	NA	NA	NA	NA	NA
55	NA	Barbados	NA	NA	NA	NA	NA
55	NA	Belarus	NA	NA	NA	NA	NA
55	NA	Belgium	NA	NA	NA	NA	NA
55	NA	Belize	NA	NA	NA	NA	NA
55	NA	Benin	NA	NA	NA	NA	NA
55	NA	Bhutan	NA	NA	NA	NA	NA
55	NA	Bolivia	NA	NA	NA	NA	NA
55	NA	Bosnia and Herzegovina	NA	NA	NA	NA	NA
55	NA	Brunei	NA	NA	NA	NA	NA
55	NA	Burkina Faso	NA	NA	NA	NA	NA
55	NA	Burundi	NA	NA	NA	NA	NA
55	NA	Cape Verde	NA	NA	NA	NA	NA
55	NA	Chad	NA	NA	NA	NA	NA
55	NA	Comoros	NA	NA	NA	NA	NA
55	NA	Congo, Dem. Republic	NA	NA	NA	NA	NA
55	NA	Congo, Republic	NA	NA	NA	NA	NA
55	NA	Cook Islands	NA	NA	NA	NA	NA
55	NA	Costa Rica	NA	NA	NA	NA	NA
55	NA	Croatia	NA	NA	NA	NA	NA
55	NA	Cuba	NA	NA	NA	NA	NA
55	NA	Cyprus	NA	NA	NA	NA	NA
55	NA	Czech Republic	NA	NA	NA	NA	NA
55	NA	Denmark	NA	NA	NA	NA	NA
55	NA	Djibouti	NA	NA	NA	NA	NA
55	NA	Dominica	NA	NA	NA	NA	NA
55	NA	East Timor	NA	NA	NA	NA	NA
55	NA	Egypt	NA	NA	NA	NA	NA
55	NA	El Salvador	NA	NA	NA	NA	NA
55	NA	Equatorial Guinea	NA	NA	NA	NA	NA
55	NA	Eritrea	NA	NA	NA	NA	NA
55	NA	Estonia	NA	NA	NA	NA	NA
55	NA	Eswatini	NA	NA	NA	NA	NA
55	NA	Fiji	NA	NA	NA	NA	NA
55	NA	Finland	NA	NA	NA	NA	NA
55	NA	Gabon	NA	NA	NA	NA	NA
55	NA	Georgia	NA	NA	NA	NA	NA

55	NA	Germany	NA	NA	NA	NA	NA
55	NA	Grenada	NA	NA	NA	NA	NA
55	NA	Guatemala	NA	NA	NA	NA	NA
55	NA	Guinea	NA	NA	NA	NA	NA
55	NA	Guinea-Bissau	NA	NA	NA	NA	NA
55	NA	Guyana	NA	NA	NA	NA	NA
55	NA	Haiti	NA	NA	NA	NA	NA
55	NA	Honduras	NA	NA	NA	NA	NA
55	NA	Hungary	NA	NA	NA	NA	NA
55	NA	Iceland	NA	NA	NA	NA	NA
55	NA	Iraq	NA	NA	NA	NA	NA
55	NA	Ireland	NA	NA	NA	NA	NA
55	NA	Japan	NA	NA	NA	NA	NA
55	NA	Jordan	NA	NA	NA	NA	NA
55	NA	Kazakhstan	NA	NA	NA	NA	NA
55	NA	Kenya	NA	NA	NA	NA	NA
55	NA	Kiribati	NA	NA	NA	NA	NA
55	NA	Kuwait	NA	NA	NA	NA	NA
55	NA	Kyrgyzstan	NA	NA	NA	NA	NA
55	NA	Lebanon	NA	NA	NA	NA	NA
55	NA	Lesotho	NA	NA	NA	NA	NA
55	NA	Liberia	NA	NA	NA	NA	NA
55	NA	Libya	NA	NA	NA	NA	NA
55	NA	Liechtenstein	NA	NA	NA	NA	NA
55	NA	Lithuania	NA	NA	NA	NA	NA
55	NA	Luxembourg	NA	NA	NA	NA	NA
55	NA	Madagascar	NA	NA	NA	NA	NA
55	NA	Malawi	NA	NA	NA	NA	NA
55	NA	Maldives	NA	NA	NA	NA	NA
55	NA	Mali	NA	NA	NA	NA	NA
55	NA	Malta	NA	NA	NA	NA	NA
55	NA	Marshall Islands	NA	NA	NA	NA	NA
55	NA	Mauritania	NA	NA	NA	NA	NA
55	NA	Mauritius	NA	NA	NA	NA	NA
55	NA	Micronesia	NA	NA	NA	NA	NA
55	NA	Monaco	NA	NA	NA	NA	NA
55	NA	Mongolia	NA	NA	NA	NA	NA
55	NA	Montenegro	NA	NA	NA	NA	NA
55	NA	Mozambique	NA	NA	NA	NA	NA
55	NA	Myanmar	NA	NA	NA	NA	NA
55	NA	Namibia	NA	NA	NA	NA	NA
55	NA	Nauru	NA	NA	NA	NA	NA
55	NA	Nepal	NA	NA	NA	NA	NA
55	NA	New Zealand	NA	NA	NA	NA	NA
55	NA	Nicaragua	NA	NA	NA	NA	NA
55	NA	Niger	NA	NA	NA	NA	NA
55	NA	Niue	NA	NA	NA	NA	NA
55	NA	North Macedonia	NA	NA	NA	NA	NA
55	NA	Norway	NA	NA	NA	NA	NA
55	NA	Oman	NA	NA	NA	NA	NA
55	NA	Palau	NA	NA	NA	NA	NA
55	NA	Palestine	NA	NA	NA	NA	NA

55	NA	Panama	NA	NA	NA	NA	NA
55	NA	Papua New Guinea	NA	NA	NA	NA	NA
55	NA	Paraguay	NA	NA	NA	NA	NA
55	NA	Portugal	NA	NA	NA	NA	NA
55	NA	Qatar	NA	NA	NA	NA	NA
55	NA	Rwanda	NA	NA	NA	NA	NA
55	NA	Saint Kitts and Nevis	NA	NA	NA	NA	NA
55	NA	Saint Lucia	NA	NA	NA	NA	NA
55	NA	Saint Vincent and the Grenadines	NA	NA	NA	NA	NA
55	NA	Samoa	NA	NA	NA	NA	NA
55	NA	San Marino	NA	NA	NA	NA	NA
55	NA	Sao Tome and Principe	NA	NA	NA	NA	NA
55	NA	Saudi Arabia	NA	NA	NA	NA	NA
55	NA	Senegal	NA	NA	NA	NA	NA
55	NA	Serbia	NA	NA	NA	NA	NA
55	NA	Seychelles	NA	NA	NA	NA	NA
55	NA	Singapore	NA	NA	NA	NA	NA
55	NA	Slovakia	NA	NA	NA	NA	NA
55	NA	Slovenia	NA	NA	NA	NA	NA
55	NA	Solomon Islands	NA	NA	NA	NA	NA
55	NA	Somalia	NA	NA	NA	NA	NA
55	NA	South Sudan	NA	NA	NA	NA	NA
55	NA	Sri Lanka	NA	NA	NA	NA	NA
55	NA	Sudan	NA	NA	NA	NA	NA
55	NA	Suriname	NA	NA	NA	NA	NA
55	NA	Sweden	NA	NA	NA	NA	NA
55	NA	Switzerland	NA	NA	NA	NA	NA
55	NA	Syria	NA	NA	NA	NA	NA
55	NA	Tajikistan	NA	NA	NA	NA	NA
55	NA	Tanzania	NA	NA	NA	NA	NA
55	NA	Togo	NA	NA	NA	NA	NA
55	NA	Tonga	NA	NA	NA	NA	NA
55	NA	Trinidad and Tobago	NA	NA	NA	NA	NA
55	NA	Tunisia	NA	NA	NA	NA	NA
55	NA	Turkey	NA	NA	NA	NA	NA
55	NA	Turkmenistan	NA	NA	NA	NA	NA
55	NA	Tuvalu	NA	NA	NA	NA	NA
55	NA	Uganda	NA	NA	NA	NA	NA
55	NA	Uruguay	NA	NA	NA	NA	NA
55	NA	Uzbekistan	NA	NA	NA	NA	NA
55	NA	Vanuatu	NA	NA	NA	NA	NA
55	NA	Vatican City	NA	NA	NA	NA	NA
55	NA	Yemen	NA	NA	NA	NA	NA
55	NA	Zimbabwe	NA	NA	NA	NA	NA

13

Table S6. World Cybercrime Index - Cashing out/money laundering

Rank	Noms	Country	I	P	TS	Overall	WCI Score
1	45	Russia	8.62	8.64	8.22	8.50	41.56
2	34	Ukraine	8.76	8.41	8.21	8.46	31.27
3	34	USA	8.15	7.00	6.47	7.21	26.63
4	29	China	8.21	7.72	7.03	7.66	24.13
5	27	UK	8.07	7.48	6.56	7.37	21.63
6	19	Nigeria	8.47	7.11	6.00	7.19	14.86
7	15	Romania	7.40	6.93	6.80	7.04	11.49
8	7	United Arab Emirates	8.14	7.29	7.29	7.57	5.76
9	7	Brazil	6.71	6.14	5.43	6.10	4.64
10	5	Germany	8.60	8.00	8.20	8.27	4.49
11	5	Poland	8.40	7.40	6.40	7.40	4.02
12	5	North Korea	7.80	7.00	6.60	7.13	3.88
13	5	Turkey	6.00	6.80	6.40	6.40	3.48
14	4	Estonia	7.75	8.25	7.25	7.75	3.37
15	5	South Africa	6.60	6.20	5.40	6.07	3.30
16	4	India	7.75	6.50	6.50	6.92	3.01
17	3	Cyprus	8.33	7.67	8.00	8.00	2.61
18	3	Ghana	10.00	8.67	5.00	7.89	2.57
19	3	Haiti	8.67	6.67	6.67	7.33	2.39
20	3	Switzerland	8.00	7.33	6.33	7.22	2.36
21	4	Philippines	5.25	5.25	4.75	5.08	2.21
21	3	Belize	7.33	6.67	6.33	6.78	2.21
23	3	Thailand	7.00	6.00	6.33	6.44	2.10
24	3	Latvia	6.33	6.00	5.33	5.89	1.92
25	2	Moldova	9.00	8.50	8.50	8.67	1.88
26	2	Myanmar	9.50	8.50	7.00	8.33	1.81
27	2	Panama	9.00	8.50	7.00	8.17	1.78
28	2	Mexico	8.50	8.00	7.50	8.00	1.74
28	2	Afghanistan	9.50	7.50	7.00	8.00	1.74
30	2	Italy	7.50	7.50	7.50	7.50	1.63
30	2	Seychelles	8.50	7.00	7.00	7.50	1.63
32	2	Netherlands	8.00	7.00	6.50	7.17	1.56
33	2	Colombia	7.50	7.00	6.00	6.83	1.49
34	3	Australia	5.67	3.67	3.33	4.22	1.38
35	2	Canada	7.00	6.00	5.50	6.17	1.34
36	2	Czech Republic	8.50	6.50	3.00	6.00	1.30
37	1	Armenia	10.00	10.00	10.00	10.00	1.09
38	1	Angola	10.00	10.00	9.00	9.67	1.05
39	1	Laos	9.00	9.00	9.00	9.00	0.98
39	1	Mozambique	10.00	8.00	9.00	9.00	0.98
41	1	Togo	10.00	10.00	5.00	8.33	0.91
42	1	Kazakhstan	8.00	8.00	8.00	8.00	0.87
42	1	Malta	8.00	8.00	8.00	8.00	0.87
44	1	Equatorial Guinea	10.00	8.00	5.00	7.67	0.83
45	1	Venezuela	9.00	6.00	7.00	7.33	0.80
45	1	Benin	10.00	5.00	7.00	7.33	0.80
47	1	Spain	7.00	7.00	7.00	7.00	0.76
48	1	Iran	7.00	6.00	7.00	6.67	0.72
48	1	Kenya	8.00	6.00	6.00	6.67	0.72

48	1	Argentina	6.00	7.00	7.00	6.67	0.72
51	1	Israel	9.00	7.00	3.00	6.33	0.69
51	1	Vietnam	7.00	6.00	6.00	6.33	0.69
53	1	Cambodia	8.00	6.00	4.00	6.00	0.65
53	1	Chile	7.00	6.00	5.00	6.00	0.65
53	1	Ecuador	7.00	6.00	5.00	6.00	0.65
53	1	Azerbaijan	9.00	6.00	3.00	6.00	0.65
53	1	Luxembourg	6.00	7.00	5.00	6.00	0.65
58	1	Malaysia	7.00	5.00	5.00	5.67	0.62
58	1	France	4.00	6.00	7.00	5.67	0.62
58	1	Sierra Leone	7.00	6.00	4.00	5.67	0.62
61	1	Bulgaria	8.00	4.00	4.00	5.33	0.58
61	1	Peru	7.00	5.00	4.00	5.33	0.58
63	1	Botswana	3.00	3.00	3.00	3.00	0.33
63	1	Ethiopia	3.00	3.00	3.00	3.00	0.33
63	1	Slovenia	4.00	3.00	2.00	3.00	0.33
63	1	Zambia	3.00	3.00	3.00	3.00	0.33
67	NA	Albania	NA	NA	NA	NA	NA
67	NA	Algeria	NA	NA	NA	NA	NA
67	NA	Andorra	NA	NA	NA	NA	NA
67	NA	Angola	NA	NA	NA	NA	NA
67	NA	Antigua and Barbuda	NA	NA	NA	NA	NA
67	NA	Austria	NA	NA	NA	NA	NA
67	NA	The Bahamas	NA	NA	NA	NA	NA
67	NA	Bahrain	NA	NA	NA	NA	NA
67	NA	Bangladesh	NA	NA	NA	NA	NA
67	NA	Barbados	NA	NA	NA	NA	NA
67	NA	Belarus	NA	NA	NA	NA	NA
67	NA	Belgium	NA	NA	NA	NA	NA
67	NA	Bhutan	NA	NA	NA	NA	NA
67	NA	Bolivia	NA	NA	NA	NA	NA
67	NA	Bosnia and Herzegovina	NA	NA	NA	NA	NA
67	NA	Brunei	NA	NA	NA	NA	NA
67	NA	Burkina Faso	NA	NA	NA	NA	NA
67	NA	Burundi	NA	NA	NA	NA	NA
67	NA	Cameroon	NA	NA	NA	NA	NA
67	NA	Cape Verde	NA	NA	NA	NA	NA
67	NA	Central African Republic	NA	NA	NA	NA	NA
67	NA	Chad	NA	NA	NA	NA	NA
67	NA	Comoros	NA	NA	NA	NA	NA
67	NA	Congo, Dem. Republic	NA	NA	NA	NA	NA
67	NA	Congo, Republic	NA	NA	NA	NA	NA
67	NA	Cook Islands	NA	NA	NA	NA	NA
67	NA	Costa Rica	NA	NA	NA	NA	NA
67	NA	Croatia	NA	NA	NA	NA	NA
67	NA	Cuba	NA	NA	NA	NA	NA
67	NA	Denmark	NA	NA	NA	NA	NA
67	NA	Djibouti	NA	NA	NA	NA	NA
67	NA	Dominica	NA	NA	NA	NA	NA
67	NA	Dominican Republic	NA	NA	NA	NA	NA
67	NA	East Timor	NA	NA	NA	NA	NA
67	NA	Egypt	NA	NA	NA	NA	NA

67	NA	El Salvador	NA	NA	NA	NA	NA
67	NA	Eritrea	NA	NA	NA	NA	NA
67	NA	Eswatini	NA	NA	NA	NA	NA
67	NA	Fiji	NA	NA	NA	NA	NA
67	NA	Finland	NA	NA	NA	NA	NA
67	NA	Gabon	NA	NA	NA	NA	NA
67	NA	Gambia	NA	NA	NA	NA	NA
67	NA	Georgia	NA	NA	NA	NA	NA
67	NA	Greece	NA	NA	NA	NA	NA
67	NA	Grenada	NA	NA	NA	NA	NA
67	NA	Guatemala	NA	NA	NA	NA	NA
67	NA	Guinea	NA	NA	NA	NA	NA
67	NA	Guinea-Bissau	NA	NA	NA	NA	NA
67	NA	Guyana	NA	NA	NA	NA	NA
67	NA	Honduras	NA	NA	NA	NA	NA
67	NA	Hungary	NA	NA	NA	NA	NA
67	NA	Iceland	NA	NA	NA	NA	NA
67	NA	Indonesia	NA	NA	NA	NA	NA
67	NA	Iraq	NA	NA	NA	NA	NA
67	NA	Ireland	NA	NA	NA	NA	NA
67	NA	Ivory Coast	NA	NA	NA	NA	NA
67	NA	Jamaica	NA	NA	NA	NA	NA
67	NA	Japan	NA	NA	NA	NA	NA
67	NA	Jordan	NA	NA	NA	NA	NA
67	NA	Kiribati	NA	NA	NA	NA	NA
67	NA	Kuwait	NA	NA	NA	NA	NA
67	NA	Kyrgyzstan	NA	NA	NA	NA	NA
67	NA	Lebanon	NA	NA	NA	NA	NA
67	NA	Lesotho	NA	NA	NA	NA	NA
67	NA	Liberia	NA	NA	NA	NA	NA
67	NA	Libya	NA	NA	NA	NA	NA
67	NA	Liechtenstein	NA	NA	NA	NA	NA
67	NA	Lithuania	NA	NA	NA	NA	NA
67	NA	Madagascar	NA	NA	NA	NA	NA
67	NA	Malawi	NA	NA	NA	NA	NA
67	NA	Maldives	NA	NA	NA	NA	NA
67	NA	Mali	NA	NA	NA	NA	NA
67	NA	Marshall Islands	NA	NA	NA	NA	NA
67	NA	Mauritania	NA	NA	NA	NA	NA
67	NA	Mauritius	NA	NA	NA	NA	NA
67	NA	Micronesia	NA	NA	NA	NA	NA
67	NA	Monaco	NA	NA	NA	NA	NA
67	NA	Mongolia	NA	NA	NA	NA	NA
67	NA	Montenegro	NA	NA	NA	NA	NA
67	NA	Morocco	NA	NA	NA	NA	NA
67	NA	Namibia	NA	NA	NA	NA	NA
67	NA	Nauru	NA	NA	NA	NA	NA
67	NA	Nepal	NA	NA	NA	NA	NA
67	NA	New Zealand	NA	NA	NA	NA	NA
67	NA	Nicaragua	NA	NA	NA	NA	NA
67	NA	Niger	NA	NA	NA	NA	NA
67	NA	Niue	NA	NA	NA	NA	NA

67	NA	North Macedonia	NA	NA	NA	NA	NA
67	NA	Norway	NA	NA	NA	NA	NA
67	NA	Oman	NA	NA	NA	NA	NA
67	NA	Pakistan	NA	NA	NA	NA	NA
67	NA	Palau	NA	NA	NA	NA	NA
67	NA	Palestine	NA	NA	NA	NA	NA
67	NA	Papua New Guinea	NA	NA	NA	NA	NA
67	NA	Paraguay	NA	NA	NA	NA	NA
67	NA	Portugal	NA	NA	NA	NA	NA
67	NA	Qatar	NA	NA	NA	NA	NA
67	NA	Rwanda	NA	NA	NA	NA	NA
67	NA	Saint Kitts and Nevis	NA	NA	NA	NA	NA
67	NA	Saint Lucia	NA	NA	NA	NA	NA
67	NA	Saint Vincent and the Grenadines	NA	NA	NA	NA	NA
67	NA	Samoa	NA	NA	NA	NA	NA
67	NA	San Marino	NA	NA	NA	NA	NA
67	NA	Sao Tome and Principe	NA	NA	NA	NA	NA
67	NA	Saudi Arabia	NA	NA	NA	NA	NA
67	NA	Senegal	NA	NA	NA	NA	NA
67	NA	Serbia	NA	NA	NA	NA	NA
67	NA	Singapore	NA	NA	NA	NA	NA
67	NA	Slovakia	NA	NA	NA	NA	NA
67	NA	Solomon Islands	NA	NA	NA	NA	NA
67	NA	Somalia	NA	NA	NA	NA	NA
67	NA	South Korea	NA	NA	NA	NA	NA
67	NA	South Sudan	NA	NA	NA	NA	NA
67	NA	Sri Lanka	NA	NA	NA	NA	NA
67	NA	Sudan	NA	NA	NA	NA	NA
67	NA	Suriname	NA	NA	NA	NA	NA
67	NA	Sweden	NA	NA	NA	NA	NA
67	NA	Syria	NA	NA	NA	NA	NA
67	NA	Tajikistan	NA	NA	NA	NA	NA
67	NA	Tanzania	NA	NA	NA	NA	NA
67	NA	Tonga	NA	NA	NA	NA	NA
67	NA	Trinidad and Tobago	NA	NA	NA	NA	NA
67	NA	Tunisia	NA	NA	NA	NA	NA
67	NA	Turkmenistan	NA	NA	NA	NA	NA
67	NA	Tuvalu	NA	NA	NA	NA	NA
67	NA	Uganda	NA	NA	NA	NA	NA
67	NA	Uruguay	NA	NA	NA	NA	NA
67	NA	Uzbekistan	NA	NA	NA	NA	NA
67	NA	Vanuatu	NA	NA	NA	NA	NA
67	NA	Vatican City	NA	NA	NA	NA	NA
67	NA	Yemen	NA	NA	NA	NA	NA
67	NA	Zimbabwe	NA	NA	NA	NA	NA

14
