# Supplementary material for: Mapping the global geography of cybercrime with the World Cybercrime Index
Source: PLoS One. 2024 Apr 10;19(4):e0297312. doi: 10.1371/journal.pone.0297312 (PMC11006133; doi:10.1371/journal.pone.0297312)
Supplement: S1 Appendix — Details of respondent characteristics and analysis of rating behaviour. (PDF) [file pone.0297312.s002.pdf]

S2 Appendix. Supporting information

Respondent nationality and residence

The survey collected data on each respondent's nationality and residence. All five major geographical regions (Africa; Asia Pacific; Europe; North America; South America) were represented in the pool of respondents (n=92). Tables S1-S4 break down the specific countries and broad regions that respondents belonged to. Respondents could only nominate a single country of nationality and a single country of residence, and were given an option of "Prefer not to say".

Table S1. Nationality (country) of respondents

Nationality	Total	% respondents
United States	24	26.09%
United Kingdom	17	18.48%
Australia	6	6.52%
Israel	4	4.35%
Nigeria	4	4.35%
Prefer not to say	4	4.35%
Netherlands	3	3.26%
Ukraine	3	3.26%
Bosnia and Herzegovina	2	2.17%
Brazil	2	2.17%
Canada	2	2.17%
Germany	2	2.17%
Poland	2	2.17%
Romania	2	2.17%
Russia	2	2.17%
Austria	1	1.09%
Benin	1	1.09%
Bulgaria	1	1.09%
Finland	1	1.09%
Gambia	1	1.09%
Ireland	1	1.09%
Italy	1	1.09%
Kenya	1	1.09%
South Korea	1	1.09%
Peru	1	1.09%
Swaziland	1	1.09%
Sweden	1	1.09%
Trinidad and Tobago	1	1.09%

Table S2. Nationality (region) of respondents

Nationality	Total	% respondents
Europe	39	42.39%
North America	27	29.35%
Asia Pacific	10	10.87%
Africa	7	7.61%
Prefer not to say	6	6.52%
South America	3	3.26%

Table S3. Residence (country) of respondents

Residence	Total	% respondents
United States	25	27.17%
United Kingdom	13	14.13%
Australia	5	5.43%
Prefer not to say	5	5.43%
Germany	4	4.35%
Israel	3	3.26%
Nigeria	3	3.26%
Switzerland	3	3.26%
Bosnia and Herzegovina	2	2.17%
Brazil	2	2.17%
Poland	2	2.17%
Russia	2	2.17%
Thailand	2	2.17%
Ukraine	2	2.17%
Bulgaria	1	1.09%
Canada	1	1.09%
China	1	1.09%
Finland	1	1.09%
France	1	1.09%
Gambia	1	1.09%
Ghana	1	1.09%
Ireland	1	1.09%
Italy	1	1.09%
South Korea	1	1.09%
Morocco	1	1.09%
Netherlands	1	1.09%
Peru	1	1.09%
Romania	1	1.09%
Singapore	1	1.09%
Spain	1	1.09%
Sweden	1	1.09%
Turkey	1	1.09%
United Arab Emirates	1	1.09%

Table S4. Residence (region) of respondents

Residence	Total	% respondents
Europe	38	41.30%
North America	26	28.26%
Asia Pacific	13	14.13%
Africa	6	6.52%
Prefer not to say	6	6.52%
South America	3	3.26%

Respondent expertise

The survey collected data about each respondent's areas of expertise. Respondents could nominate themselves as experts in any of the five cybercrime types (Technical products/services; Attacks and extortion; Data/identity theft; Scams; Cashing out/money laundering) and/or any of the five geographical regions (Africa; Asia Pacific; Europe; North America; South America), including an option of "Other". Tables S5-S6 show the number of respondents who nominated themselves as experts, and what they were experts in.

Respondents were often experts in multiple cybercrime types and regions. The majority of respondents (n=84) nominated themselves as an expert in a particular type of cybercrime. Fewer respondents (n=50) nominated themselves as experts in a particular region. Respondent expertise is not mutually exclusive; respondents could nominate themselves as experts in multiple cybercrime types as well as multiple regions. Each Table specifies the number of experts

Table S5. Expertise - cybercrime type

Expertise	Total	% type experts (n=84)	% all respondents
Attacks and extortion	55	63.95%	59.78%
Data and identity theft	53	61.63%	57.61%
Cashing out and money laundering	45	52.33%	48.91%
Scams	45	52.33%	48.91%
Technical products and services	45	52.33%	48.91%
Other	16	18.60%	17.39%
None	8	9.30%	8.70%

Table S6. Expertise - region

Expertise	Total	% region experts (n=50)	% all respondents
None	42	84.00%	45.65%
Europe	28	56.00%	30.43%
Asia Pacific	15	30.00%	16.30%
Africa	12	24.00%	13.04%
South America	6	12.00%	6.52%
North America	3	6.00%	3.26%
Other	1	2.00%	1.09%

Intra-individual response variability

To test the effect of response fatigue in our data, we produced an intra-individual response variability (IRV) index, which calculates the standard deviation of consecutive scores given by a respondent. The more strings of repeating scores, the lower the participant's IRV; an IRV of 0 would signify that the respondent gave the same score for each question.

Table S7 summarises the IRV distribution across the survey. Table S8 lists the full IRV index. In both Tables, columns 1-5 designate each cybercrime type in the order they appeared in the survey. The cybercrime types appeared in this order: 1) *Technical products/services*, 2) *Attacks and extortion*, 3) *Data/identity theft*, 4) *Scams*, and 5) *Cashing out/money laundering*. NA designates that the participant skipped the section.

Table S7. Summary of IRV distribution in each section of the survey

	minimum	Q1	median	Q3	maximum
**1 Technical products/services**	0.000000	0.983165	1.354005	1.808628	2.987000
**2 Attacks and extortion**	0.000000	0.816497	1.238272	2.063285	2.971000
**3 Data/identity theft**	0.000000	0.990430	1.334523	1.919821	2.968000
**4 Scams**	0.000000	0.971825	1.424001	1.951800	3.079000
**5 Cashing out/money laundering**	0.000000	0.822288	1.164500	1.747943	3.100000

Table S8. Full IRV index

ID	1	2	3	4	5	Overall
1	1.290994	1.164500	1.302013	0.927961	1.861899	1.329490
2	0.752773	0.632456	0.752773	NA	0.516398	1.007220
3	1.424001	0.816497	1.407463	1.585923	1.497473	1.741607
4	0.971825	0.781736	0.707107	2.094365	1.162919	1.680342
5	2.874918	1.585923	2.968267	3.078518	0.516398	2.472843
6	2.380476	2.354327	1.919821	3.058166	1.934647	2.671640
7	2.455153	1.366260	2.153624	2.000000	1.643168	1.969744
8	1.597617	1.060099	1.069045	1.624221	1.830951	1.541966
9	0.516398	0.577350	0.881917	0.983192	0.577350	1.110684
10	0.937437	0.000000	NA	NA	1.128152	1.008014
11	1.099784	0.743223	0.828079	0.833333	0.828079	0.864424
12	0.668558	1.302013	1.187234	2.554175	2.722044	1.996035
13	1.641718	1.224745	1.496026	1.424001	1.676486	1.792640
14	1.060099	1.533747	1.245946	1.060099	1.302013	1.378732
15	1.676163	2.877113	1.288057	0.707107	0.000000	1.785357
16	2.093072	1.118034	0.408248	1.471960	0.816497	1.714358
17	1.927248	2.642149	1.437591	2.231805	2.484236	2.363994
18	2.059715	1.154701	1.563472	1.473577	0.985611	1.621398
19	0.996205	0.743223	1.407463	1.187234	1.505545	1.735887
20	2.256304	2.645751	NA	NA	NA	2.372461
21	0.975900	1.407463	0.703732	1.463850	1.032796	1.244809
22	0.333333	0.577350	0.500000	0.516398	0.717741	1.518220
23	2.016598	1.681836	1.927248	1.690309	1.882248	1.898601
24	0.887625	1.302678	1.290994	1.311372	1.454058	1.372473
25	1.787301	1.092906	1.269296	2.260777	1.763834	1.616518
26	1.959106	2.405801	2.416215	1.812654	1.355764	2.129558
27	1.486447	0.500000	2.153624	2.886751	1.732051	2.087723
28	2.288689	2.177810	1.851640	1.638815	2.138090	2.009258
29	0.000000	1.971222	2.691175	2.160247	0.000000	2.360876
30	2.000000	1.414214	2.048034	1.333333	0.881917	2.076808
31	0.000000	1.095445	2.000000	1.732051	NA	2.104417
32	1.505545	0.915475	0.899735	1.334523	0.703732	1.187472
33	1.302013	1.505545	1.362770	2.574643	1.944467	1.787050
34	1.138180	1.471960	1.166667	1.691482	2.336053	1.716586
35	1.851640	0.577350	0.547723	0.577350	0.547723	2.194173
36	0.632456	1.536591	1.333333	0.632456	1.211060	1.704103
37	1.362770	2.699206	1.234427	2.000000	1.709915	1.933217
38	1.224745	0.816497	NA	NA	NA	1.060099
39	0.736788	1.060099	0.414039	0.985611	0.990430	0.902848
40	3.109126	2.585675	2.716791	3.023716	3.000000	3.024644
41	1.387015	1.642245	1.370689	1.370689	1.763834	1.520110

42	1.000000	1.000000	1.404076	0.500000	1.044466	1.160134
43	1.373213	1.521278	1.032796	0.990430	1.309307	1.533764
44	1.234427	1.387015	1.125463	0.883715	0.676123	1.131052
45	2.642149	2.386833	2.146173	1.878238	1.164500	2.550008
46	2.015095	1.869556	2.796101	2.778434	2.574643	2.370561
47	1.624221	1.169045	1.162919	0.577350	1.029857	1.592138
48	1.471960	0.577350	1.527525	0.000000	0.000000	2.275186
49	1.759329	1.481366	2.445599	2.179449	2.061553	2.346773
50	0.500000	0.258199	1.527525	0.834847	0.774597	1.198651
51	1.060099	1.242118	0.798809	0.941124	1.099784	2.084520
52	0.389249	0.000000	0.507093	0.000000	0.516398	0.504349
53	1.681836	1.162919	1.437591	1.502379	1.055597	1.382907
54	2.987275	2.635231	3.752777	4.647580	4.226898	3.512326
55	1.641718	2.160247	1.767430	1.322876	2.126925	2.482762
56	0.975900	0.668558	0.666667	0.971825	0.927961	0.867336
57	4.131182	1.121224	2.631313	0.816497	1.463850	2.825049
58	1.352247	1.807392	1.099784	1.048809	NA	1.417814
59	0.752773	1.322876	2.088932	NA	1.032796	1.586520
60	0.883715	0.816497	0.883715	0.703732	0.866025	0.890636
61	1.667619	2.416215	2.006932	2.886751	NA	2.089879
62	1.732051	1.882938	1.900292	1.900292	1.355764	1.749114
63	1.825742	2.291288	1.549193	1.804756	1.083625	2.213282
64	1.095445	1.201850	1.230915	1.000000	0.666667	1.173357
65	1.666667	2.354879	1.154701	1.527525	2.756810	2.128451
66	2.108185	2.229282	1.112697	NA	NA	1.763834
67	0.915475	0.000000	0.000000	1.000000	0.983192	1.156690
68	1.309307	0.651339	0.440959	0.333333	NA	0.920035
69	0.000000	0.000000	0.000000	2.516611	0.000000	1.102962
70	2.472708	2.587516	2.319688	2.068279	2.081666	2.499044
71	0.723747	NA	0.000000	0.866025	0.632456	0.716746
72	1.014185	1.207122	1.397276	1.125463	1.641718	1.388498
73	1.146423	1.234427	0.990430	1.579632	1.387015	1.304877
74	0.000000	0.408248	2.305273	2.028370	2.028370	2.085275
75	1.552264	1.000000	0.971825	0.753778	0.674200	1.154701
76	1.533747	2.063284	1.099784	1.951800	1.242118	1.824557
77	2.097618	2.971291	2.193063	1.709915	1.597617	2.232278
78	1.032796	0.883715	1.146423	1.521278	0.703732	1.092480
79	1.083625	NA	0.632456	NA	NA	0.978528
80	1.242118	0.743223	1.290994	0.000000	1.772811	1.853849
81	0.797724	1.099784	1.579632	0.639940	0.000000	1.018510
82	0.798809	1.187234	0.703732	0.593617	0.703732	0.833315
83	2.443651	2.282438	2.642149	1.791514	0.833809	2.105334
84	1.146423	1.222799	1.334523	1.060099	1.082326	1.185954
85	1.264911	0.600925	0.816497	1.000000	0.440959	1.319029
86	1.032796	0.915475	1.334523	1.290994	0.915475	1.159838
87	1.791514	2.345208	2.483277	1.878238	NA	2.223661
88	1.355764	0.755929	1.114641	1.311372	1.146423	1.157834
89	0.990430	2.111195	1.767430	2.229670	1.698739	1.823273
90	2.035401	2.261479	1.355764	1.320173	1.632993	1.847521
91	1.643168	2.236068	3.976119	3.618734	3.100179	3.204698
92	1.320173	2.000000	1.812654	1.397276	1.215431	1.610644
