# Supplementary material for: Mapping the global geography of cybercrime with the World Cybercrime Index
Source: PLoS One. 2024 Apr 10;19(4):e0297312. doi: 10.1371/journal.pone.0297312 (PMC11006133; doi:10.1371/journal.pone.0297312)
Supplement: Attachment — Submitted filename: brucerev.pdf [file pone.0297312.s003.pdf]

Reviewer's Report:

Mapping the Global Geography of Cybercrime with the World Cybercrime Index

PLOS ONE manuscript ID PONE-D-23-32959

1. 269-271: How long is the survey? As you know, there is a literature on respondent fatigue when answering a long survey. Please reference this literature and explain in more detail why you don't believe that respondents resorted to random answers due to fatigue. See for example, https://www.nber.org/papers/w30439.

2. How accurate are your cybercrime professionals at correctly identifying the physical location of cybercriminals? I think you need to provide some measure of this accuracy rather than simply relying on the reputation of your respondents because, as you state, correctly identifying the physical location of a cyberattack's author is very difficult given the nearly untraceable nature of internet traffic.

3. 350-363: The manuscript implicitly assumes all cybercrime is conducted by non-state actors: either individuals or by members of criminal networks (organized crime). But it is well-known that many cybercriminals are employed and supported within state-sponsored facilities. Examples include Russia and North Korea. Please explain how your WCI could be used to reduce the amount of cybercrime generated by this population.

4. 350-363: Many of the discussed approaches to fighting cybercrime implicitly assume these criminals reside in developing countries. But the four top offenders, Russia, China, Ukraine, and the United States are developed countries. What strategies that use your WCI might be effective at turning cybercriminals in these countries away from cybercrime? Specifically, many of these individuals are educated and fully employed.

5. There are no equation numbers.
